# Study on Self-Healing Effect of Concrete Based on Epoxy Resin Adhesive

**DOI:** 10.3390/ma18122679

**Published:** 2025-06-06

**Authors:** Jianguo Lv, Shenlong Niu, Wei Zhang, Yongshuai Sun

**Affiliations:** 1 College of Engineering and Technology, China University of Geosciences, Beijing 100083, China; lvjianguo6662023@163.com (J.L.); niushenong666@163.com (S.N.); 2 School of Earth Science and Surveying Engineering, China University of Mining and Technology, Beijing 100083, China; zwandbetsy@126.com; 3 College of Water Resources and Civil Engineering, China Agricultural University, Beijing 100083, China

**Keywords:** self-healing concrete, epoxy resin adhesive, hollow glass tubes, mechanical properties, healing efficiency

## Abstract

The issues concerning the stability and durability of concrete structures have garnered increasing attention. This study, through theoretical analysis and experimental investigation, explores the application of epoxy resin adhesives in self-healing concrete. A three-point bending test was conducted on the self-healing system, employing C30 concrete as the substrate, with glass double tubes 80 mm in length and 1 mm in wall thickness, possessing an inner diameter of 10 mm, serving as the resin reservoirs, and modified epoxy resins serving as the healing agents. The experimental data indicate that the healing system has a certain degree of strength recovery effect on the concrete matrix, yet the recovery rate is suboptimal, and there are areas for improvement. Furthermore, this paper investigates the optimization of the epoxy resin adhesive formulation and its efficacy in self-healing concrete applications.

## 1. Introduction

Concrete is a kind of brittle heterogeneous body that easily produces large deformations and diplays cracking under external loads. The causes of concrete cracks are raw materials, improper construction, temperature, drying shrinkage, uneven settlement, and local stress [1,2,3,4,5]. Specifically, concrete cracks can be divided into microcracks and macrocracks. At present, the research and control objects of the academic and engineering circles are mainly macroscopic cracks [6]. When the concrete structure is subjected to dynamic or static stress, microcracks will occur. If the load value exceeds the critical strength of the concrete, microcracks will gradually develop into macroscopic cracks [7,8,9,10]. In addition, the destructive force generated by natural disasters can also cause cracks in the concrete [11,12].

Nowadays, the method of concrete crack repair in the engineering field is mainly passive and artificial post-repair. There are many kinds of repair methods and equipment, and these mainly address the causes of cracking, the shape of cracks, the function and importance of the building structure, and the surrounding environment. The common methods of concrete crack repair include structural reinforcement, surface treatment, grouting, filling, and so on [13,14]. In addition, with the development of the quality of life, people’s safety awareness is also increasing, and the requirements for stable and safe engineering structures are more stringent. To a certain extent, this passive and artificial post-repair mode is unable to meet the requirements of the modern construction industry for concrete materials. Therefore, the research and preparation of self-healing concrete and self-healing technology are attracting more and more attention around the world [15]. To solve these problems, scientists propose the concepts of the self-diagnosis and self-healing of material damage. Recently, academic circles studied and established a variety of different concrete crack self-healing technologies. These mainly include polymer curing technology [16,17,18], crystallization precipitation technology [19,20], osmotic crystallization precipitation technology [21,22,23,24], and microcapsule technology [25,26,27,28,29]. In addition, the use of microorganisms for repair [19,30,31,32,33] is also one of the hot topics. Shadhar et al. [34] utilized microbial methods for the self-healing of concrete, exploring the effects of different environmental conditions on the healing efficacy. Zheng et al. [35], on the other hand, investigated the impact of silica fumes on the self-healing behavior of Ultra-High-Performance Fiber-Reinforced Concrete (UHPFRC) from the perspective of material composition, revealing the relationship between hydration processes and structural regeneration. Lefever et al. [36] assessed self-healing cementitious materials using ultrasonic technology, offering a novel non-destructive method for evaluating the self-healing effects. Alshalif et al. [37] focused on optimizing the self-healing of bio-foamed concrete, providing new ideas for the development of eco-friendly construction materials.

Epoxy resin adhesives, known for their excellent adhesion, mechanical properties, and chemical stability, are considered effective self-healing materials [38]. However, the high viscosity of epoxy resins and the volume shrinkage during curing limit their application in self-healing concrete [39]. To address this issue, researchers optimized the performance of epoxy resin adhesives by adding active diluents and modifying curing agents. Hollow glass tubes, as a novel type of self-healing container, are widely used to encapsulate healing agents due to their good chemical stability and mechanical properties [40]. By rationally selecting the size and arrangement of hollow glass tubes, the self-healing effect of concrete can be significantly improved.

**Figure 1:**
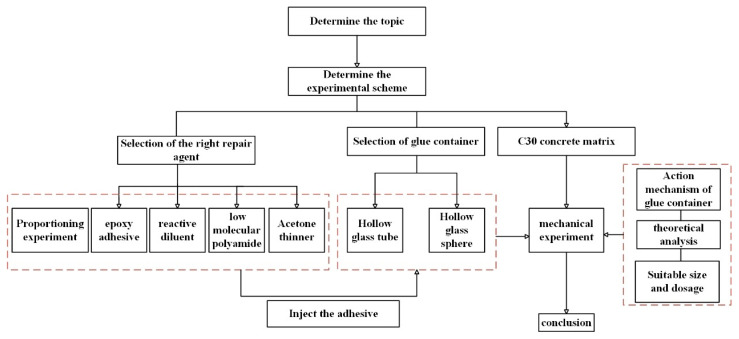
Technology roadmapping.

This study aims to explore the application effects of epoxy resin adhesives in self-healing concrete through theoretical analysis and experimental research. By optimizing the formulation and process, the fluidity and permeability of the repair agent are enhanced to effectively repair concrete cracks. At the same time, the feasibility of using hollow glass tubes as a reservoir for the repair agent is investigated, providing theoretical basis and technical support for the development of self-healing concrete technology. The main structure of the article is shown in Figure 1.

## 2. Experimental Overviews

### 2.1. Formulation of Epoxy Resin Adhesives

For the self-healing mechanism within self-repairing concrete structures, the adhesive selected must satisfy specific conditions: it should exhibit strong adhesion and good fluidity, with curing conditions that are straightforward and do not require intricate or specialized operational techniques [41]. The incorporation of the adhesive should not compromise the system’s inherent properties, featuring minimal volume shrinkage, moderate curing times, and enduring chemical stability. It is storable in a resin reservoir without degradation. In theoretical assessments, adhesives with a multitude of components tend to exhibit stable performance, and the greater the number of components, the better their shelf life and temperature adaptability. Considering these factors, this study opts for a multi-component epoxy resin adhesive as the healing agent for experimentation, selecting common E-51 epoxy resin, 692 reactive diluent, 650 low-molecular-weight polyamide curing agent, and acetone in nine distinct mixture ratios. The experimental research is conducted across three dimensions—bonding strength, fluidity, and curing time—to identify the optimal combination that aligns with the comprehensive performance demands of the experiment.

#### 2.1.1. Adhesive Formulation Ratios

This study utilizes 692 epoxy resin as a reactive diluent. By adjusting the amount of the diluent, the viscosity of the epoxy resin can be reduced to a reasonable range. Moreover, due to the presence of the epoxy groups in the reactive diluent, which participate in the curing reaction with the curing agent, the 650 polyamides cannot be diluted with the reactive diluent. Instead, a non-reactive diluent should be used for dilution. In this study, acetone, which lacks epoxy groups, is selected. During the experiments, using a ratio of epoxy resin to 692 diluents of approximately 1:0.2 and a ratio of 650 low-molecular-weight curing agent to acetone of approximately 1:0.3 resulted in good fluidity. The specific optimized formulation ratios need to be further determined through orthogonal experiments and tests with a six-speed rotational viscometer. The calculation formula for the amount of amine curing agent is as follows:

(1) W=MHn×E

where W is the mass of the curing agent required for 100 g of epoxy resin, in grams (g). MHn is the equivalent of active hydrogen of the curing agent. E is the epoxy value of the epoxy resin.

The epoxy value of E-51 epoxy resin is approximately 0.50 to 0.55, and the equivalent of active hydrogen for the 650 low-molecular-weight polyamide curing agent is about 140. From this, it can be roughly calculated that the amount of curing agent required for 100 g of E-51 epoxy resin is approximately 80 g. Additionally, there is no strict requirement for the amount of curing agent added; it can be adjusted according to specific requirements. In experiments, the epoxy groups in the epoxy resin reactive diluent participate in the curing reaction. Theoretically, this allows for an increase in the amount of curing agent. In practice, the amount of curing agent required for every 100 g of epoxy resin is determined to be 90 to 110 g.

#### 2.1.2. Determination of Adhesive Formulation Ratios via Orthogonal Experiments

The experimental protocol of this study, adapted to practical engineering, involves incorporating 692 reactive diluents, 650 curing agents, and acetone, as a non-reactive diluent, into the epoxy resin. This is followed by thorough mixing to ensure uniformity. The factors under examination are threefold, with three levels selected for each. Thus, employing an L9 (3 × 3) orthogonal array for the experimental design, which is shown in Table 1. With 100 g of epoxy resin as the reference, the quantities of 692 reactive diluent range from 15 to 25 g, the quantities of 650 curing agent range from 90 to 110 g, and the quantity of acetone ranges from 25 to 35 g. Various ratios of the repair agent system are formulated, and the repair agent is applied to the cracks in cement mortar. After complete curing, a three-point bending test is executed to ascertain the flexural strength, thereby identifying the optimal formulation ratio for the repair agent.

#### 2.1.3. Adhesive Flowability Test

**Figure 2:**
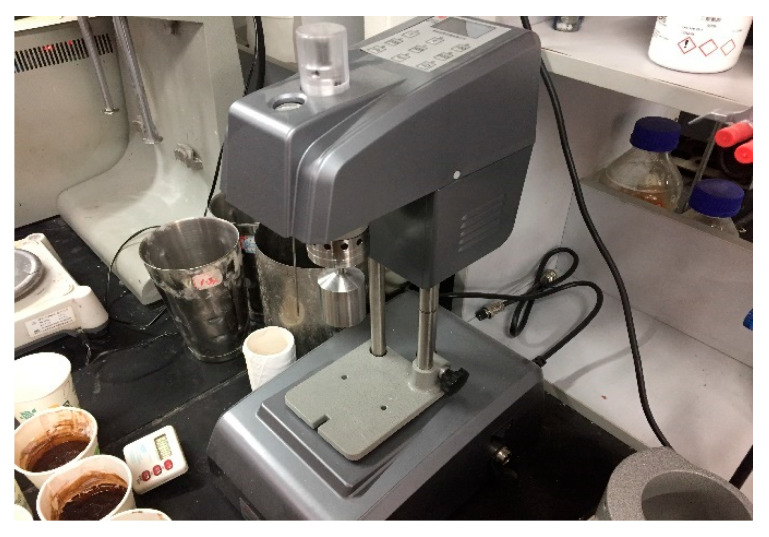
Six-speed rotary viscometer.

Optimal formulation ratios are ascertained through viscosity testing using a six-speed rotational viscometer: Rotate the outer cylinder counterclockwise to detach it. Unscrew the inner cylinder counterclockwise and gently push upwards to align it with the conical end of the inner cylinder shaft. Exercise care to prevent deformation or damage to the inner cylinder shaft. Rotate the outer cylinder clockwise to secure it in place. At 3 revolutions per minute (r/min), ensure there is no oscillation of the outer cylinder; if oscillation occurs, cease operation and reposition the outer cylinder. The display of the instrument is shown in Figure 2. At 3 r/min, verify that the pointer on the dial does not deviate; if it is not at the zero mark, calibration is necessary. Pour the freshly mixed adhesive into the sample cup up to the indicated line, promptly place it on the tray, and elevate the tray to align the liquid surface in the inner cup with the mark on the outer cylinder. Once the reading on the dial stabilizes, record the measurement.

#### 2.1.4. Adhesive Curing Time

Typically, undiluted and unmodified epoxy resin adhesives can fully cure at room temperature within 24 h of being mixed with a curing agent. To investigate the effect of the non-reactive diluent acetone on the curing of epoxy resins, a control group was established in the experiment, with varying proportions of acetone added sequentially. The specific formulations of the adhesives for each group are shown in Table 2. Curing tests were conducted for the formulations listed in Table 2, and the complete curing times for each group of adhesives were recorded.

### 2.2. Selection of Adhesive Storage Containers

In this study, the self-healing technology for concrete cracks utilizes hollow glass tubes as glue storage vessels. In this section, based on composite material theory, a mechanical analysis model of the interaction between the glass tubes in the concrete and the structural components is established. Zhao Guofan et al. [42] made the following assumptions for the composite material model: the glass tubes are continuously and uniformly arranged parallel to each other and in the direction of the applied force; the glass tubes are perfectly bonded to the matrix, exhibit elastic deformation with the matrix, and have equal lateral deformation. According to the basic assumptions of the composite model, using the hollow glass tube as a resin container for the analysis model, the material stress formula for this type of self-healing concrete is derived as follows:

(2) $$\begin{array}{c} \sigma =\rho_{a}\sigma_{a}+\rho_{b}\sigma_{b} \end{array}$$

where σ is the average stress of the composite material; $\rho_{a}$ is the stress of the self-healing glass tubes; $\sigma_{a}$ is the volume fraction of the glass tubes; $\rho_{b}$ is the stress of the matrix; and $\sigma_{a}$ is volume fraction of the matrix.

Due to the fundamental properties of glass tubes influencing the performance of both the glass tubes and the matrix, it is necessary to make corrections to Equation (2). The factors typically considered for these corrections include the effective coefficient for the irregular distribution of glass tubes, the length coefficient of the glass tubes, and the interfacial bonding between the glass tubes and the matrix. The orientation factor $\eta_{\theta }$ can be calculated using the equip–inclination theory, which is the average ratio of the projected length of the glass tubes in a specific direction compared to the length of the glass tubes within the concrete. For a glass tube of length $l_{f}$ oriented in a three-dimensional coordinate system, according to the equip–inclination theory, the probability of the tube’s horizontal angle θ and vertical angle φ being equal can be used to calculate the orientation factor of the glass tube in any direction in three dimensions using Equation (3):

(3) $$\begin{array}{c} \eta_{\theta }=\frac{\int_{0}^{\frac{\pi }{2}}\int_{0}^{\frac{\pi }{2}}\cos\theta \cos\varphi \;d\theta \;d\varphi }{{\left(\frac{\pi }{2}\right)}^{2}}\approx 0.41 \end{array}$$

To analyze the influence of the length of glass tubes, length coefficient $\eta_{l}$ is introduced. Taking an individual glass tube as the subject of study, when concrete undergoes cracking, the equiprobability theory is still adopted. Assuming that the probability of the glass tube intersecting with the crack at any point is the same, then the length coefficient is valued at $\eta_{l}=0.5$. Considering the orientation and length factors of the glass tubes comprehensively, an effective coefficient for the regular distribution of the glass tubes is introduced. The stress formula for concrete incorporating irregularly distributed glass tubes can be derived as follows:

(4) $$\begin{array}{c} \sigma =\left(1-\rho_{b}\right)\sigma_{a}+\eta_{\theta }\eta_{1}\rho_{b}\sigma_{b} \end{array}$$

The incorporation of glass tubes must be strictly controlled: too little will not achieve the self-healing effect, while too much will affect the inherent properties of the concrete. Therefore, the parameter of the critical volume fraction of glass tubes has been proposed. This parameter refers to the minimum volume fraction of glass tubes at which the concrete material can still carry a load without a decrease in capacity after cracks have formed. The calculation of the stress intensity of the composite is shown in Equation (5):

(5) $$\begin{array}{c} \sigma_{c}=\left(1-\rho_{bc}\right)\sigma_{ac}+\eta_{\theta }\eta_{1}\rho_{bc}E_{b}\varepsilon_{ac} \end{array}$$

where $\sigma_{ac}$ represents the ultimate strength of the matrix, $\varepsilon_{ac}$ represents the ultimate strain of the matrix, and $E_{b}$ is the elastic modulus of the glass tubes. After the matrix cracks, the unloading is borne by the glass tubes. If $\sigma_{ac}=0$, then the equation becomes the following:

(6) $$\begin{array}{c} \sigma_{bc}=\eta_{\theta }\eta_{1}\rho_{bc}\sigma_{bc} \end{array}$$

where $\sigma_{bc}$ represents the stress intensity of the glass tube. When $\sigma_{bc}<\sigma_{c}$, it indicates that after the composite material cracks, the embedded glass tubes do not have sufficient strength to bear the increased load due to the unloading of the matrix, leading to a state of failure where the glass tubes may be pulled out or break. When $\sigma_{bc}>\sigma_{c}$, it suggests that after the matrix cracks, the load borne by the matrix is transferred to the glass tubes. At this point, the glass tubes bear the entire load applied to the component, and they may also be pulled out or break due to the significant load. When $\sigma_{bc}=\sigma_{c}$, the critical volume fraction of the glass tubes can be determined as follows:

(7) $$\begin{array}{c} \rho_{bc}=\frac{\sigma_{ac}}{\left(\sigma_{bc}-E_{b}\varepsilon_{ac}\right)\eta_{\theta }\eta_{l}+\sigma_{ac}} \end{array}$$

**Figure 3:**
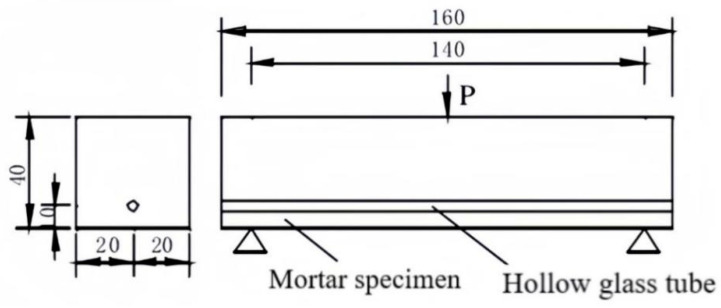
Mortar test blocks.

Based on this premise, this section carries out experiments on 12 cement mortar specimens, 9 of which are embedded with hollow glass tubes, while the other 3 are conventional mortar flexural specimens. The specific mix proportions of the cement mortar are detailed in Table 3:

The dimensions of the mortar specimens are 40 mm × 40 mm × 160 mm. The inner diameter of the hollow glass tubes is 10 mm, with a wall thickness of 1 mm and a length of 40 mm. The positions of their embedding are illustrated in Figure 3.

### 2.3. Self-Healing Effect Inspection

#### 2.3.1. Experimental Materials

**Figure 4:**
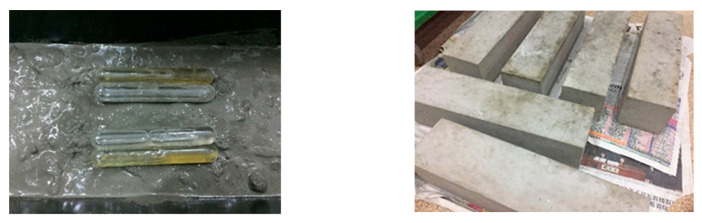
Self-healing concrete test blocks.

**Figure 5:**
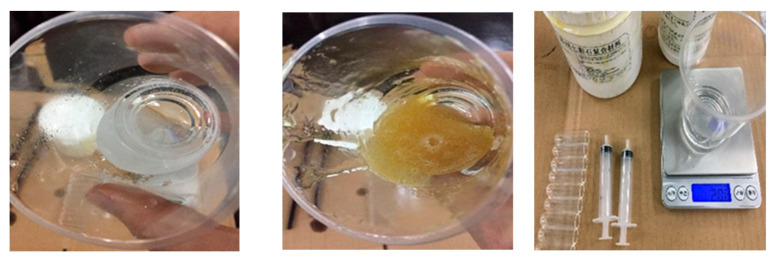
Adhesive.

The concrete specimens fabricated for this experiment had dimensions of 100 mm × 100 mm × 400 mm and were made of C30-grade concrete. C30 concrete is widely applicable in various engineering projects, including housing, roads, bridges, and tunnels. The cement used is ordinary Portland cement with a strength grade of 42.5. The mix proportions are detailed in the Table 4:

Concrete specimens for this experiment were mixed using a hand-push mixer. Coarse aggregate, cement, and sand were sequentially poured into the mixer and dry-mixed for 90 s to ensure a uniform blend. Subsequently, water was added while continuing to mix for an additional 150 s until a homogeneous mixture was achieved, at which point casting commenced. Due to the incorporation of hollow glass tubes into the self-healing concrete specimens, conventional casting methods for ordinary concrete were not suitable for this experiment. Therefore, a layered casting method was introduced for the pouring process. After casting, the perimeter was compacted with a trowel, and the surface was smoothed. The specimens were then left to rest for 24 h before demolding and placed in a curing room for standard curing until they reached an age of 7 days, after which a three-point bending test was conducted. The concrete specimens are as follows and Figure 4 and Figure 5 display the self-healing concrete test blocks and the adhesive. In the third picture of the Figure 5, the bottle on the left is 650 low molecular weight polyamide in acetone, and the bottle on the right is 692 reactive diluent:

1. Three C30 concrete specimens with dimensions of 400 mm × 100 mm × 100 mm.
2. Three C30 concrete specimens with built-in self-healing systems, each with dimensions of 400 mm × 100 mm × 100 mm.

#### 2.3.2. Determination of Storage Container Parameters for Adhesive

(8) $$\begin{array}{c} \rho_{bc}=\frac{\sigma_{ac}}{\left(\sigma_{bc}-E_{b}\varepsilon_{ac}\right)\eta_{\theta }\eta_{l}+\sigma_{ac}} \end{array}$$

where $\sigma_{ac}$ is the tensile strength of C30 concrete, taken as 2.01  MPa; $\sigma_{bc}$ is the ultimate tensile strength of glass, taken as 90  MPa; $E_{b}$ is the elastic modulus of glass, taken as 55  GPa; and $\varepsilon_{ac}$ is the ultimate tensile strain of concrete, taken as 0.0001.

When $\eta_{\theta }$ is taken as 0.64, $\rho_{bc}$ is approximately equal to 7%. Subsequent experiments on the influence of different volume fractions of glass tubes on the flexural strength of concrete determined that the most suitable volume fraction of glass tubes is around 2%.

(9) $$\begin{array}{c} L_{cr}=\frac{\sigma_{F}}{\tau_{y}}t \end{array}$$

where $L_{cr}$ represents the critical length of the glass tube; $\sigma_{F}$ is the ultimate tensile strength of the glass tube, taken as 90  MPa; $\tau_{y}$ is the yield shear stress of the concrete matrix, taken as 2.01  MPa; t the wall thickness of the glass tube, derived using Equations (2)–(10).

(10) $$\begin{array}{c} t=\frac{\left(2.7C+0.1\frac{d}{\rho_{te}}\right)\nu \tau_{y}}{1.5\sigma_{F}} \end{array}$$

The calculation yields a value of approximately 1 mm for t. The final results indicate that $L_{cr}$ is approximately 40 mm. It was observed in the experiments that glass tubes of 40 mm in length are less prone to cracking. Consequently, to further optimize the system, we selected glass tubes of 80 mm in length.

#### 2.3.3. Compressive Strength Test

According to the formula, by comparing the flexural (compressive) strength of concrete specimens with embedded gel-storage containers and those without (plain concrete specimens), the flexural (compressive) strength loss rate is introduced as a quantitative indicator to evaluate the impact of the gel-storage container on the strength of the concrete matrix. The calculation formula is as follows:

(11) $$f_{a}=\frac{f-f’}{f}\times 100\%$$

where:

$f_{a}$—Flexural (compressive) strength loss rate (%)

f—Flexural (compressive) strength of plain concrete specimens (MPa)

f’—Flexural (compressive) strength of concrete specimens with embedded glass spheres (MPa)

Using the Jinan Shijin WDW-200E microcomputer-controlled electronic universal testing machine (Jinan, China), which has a maximum testing force of 200 kN and is equipped with both compression and bending fixtures, the equipment offers high precision and responsive control, making it suitable for the mechanical performance testing of small concrete and mortar specimens. During the experiment, 100 mm × 100 mm × 100 mm concrete cubes are placed in the compression fixture and subjected to compressive testing at a loading rate of 0.5–1.0 MPa/s. Meanwhile, 40 mm × 40 mm × 160 mm mortar specimens are placed in a three-point bending fixture, loaded to failure at the specified span and loading rate, and the maximum load is recorded to calculate the strength.

The porosity was quantified in terms of the volumetric ratio of glass spheres, and the critical volume content can be calculated using the formula fp2/3 = 1 − S/S0. The influence of the addition of glass spheres on the strength of the concrete matrix is dual and cannot be generalized. Considering only the negative impact of pre-formed pores, based on the assumption that the theoretical strength loss of concrete does not exceed 15% (Table 5), the critical volume content of glass spheres is calculated to be 5.8%.

Pre-formed pores: Concrete typically has a connected porosity rate of less than 5%, but it is difficult to measure this precisely. Here, concrete without glass spheres is idealized as a homogeneous matrix with 0% porosity, and pores are pre-formed at rates of 0%, 2%, 4%, and 6%.

**Figure 6:**
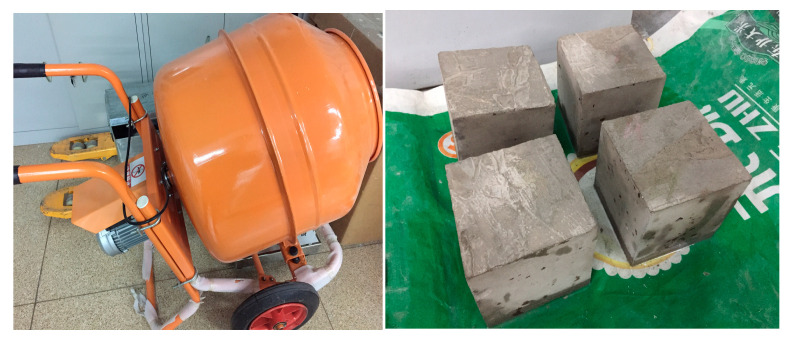
Compressive test specimens.

Four groups of C30 concrete specimens (100 mm × 100 mm × 100 mm cubes for compressive testing) were prepared (Figure 6):

1. No glass spheres;
2. Glass sphere content of 2% (40 spheres with 10 mm inner diameter);
3. Glass sphere content of 4% (80 spheres with 10 mm inner diameter);
4. Glass sphere content of 6% (120 spheres with 10 mm inner diameter).

After 7 days of curing, the specimens were subjected to a compression test, and the results are presented in Table 6.

**Figure 7:**
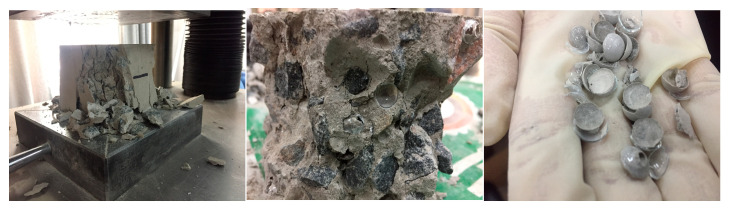
Failure surface.

Results were analyzed, and the results are shown in Figure 7:

1. The addition of glass spheres does affect the compressive strength of the concrete matrix. The weakening effect outweighs any reinforcement, resulting in a certain loss of strength. However, the influence is minor when the content is below 6% (Table 6).
2. The theoretical strength loss rate is higher than the experimental values, possibly because the prediction model does not account for the potentially positive effects of the glass material on concrete strength.

#### 2.3.4. Flexural Strength Test

**Figure 8:**
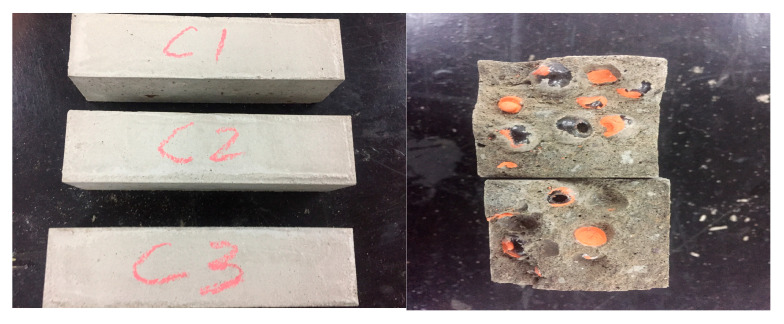
Flexural test specimens and their failure surfaces.

Four groups of cement mortar specimens (40 mm × 40 mm × 160 mm beams for flexural testing) were prepared:

1. No glass spheres;
2. Glass sphere content of 2% (Figure 8C1);
3. Glass sphere content of 4% (Figure 8C2);
4. Glass sphere content of 6% (Figure 8C3).

Table 7 presents the experimental results of flexural strength.

1. The presence of glass spheres clearly weakens the flexural strength of the concrete matrix, likely because the glass spheres themselves have poor flexural performance. When the number of spheres is high, the overall flexural strength of the specimen is significantly limited.
2. From the failure surfaces, it is evident that the glass spheres were largely undamaged, indicating that cracks did not penetrate the spheres. Therefore, using glass spheres as encapsulating containers is not recommended.

#### 2.3.5. Experimental Procedure

(1) After being cured under standard conditions for a 7-day period, the surface of the concrete specimens is wiped clean and marked.
(2) During the three-point bending test of concrete specimens, the load is applied uniformly and continuously until the specimen fails and a crack of approximately 3 mm width appears, at which point the test is halted. The failure load and the location of the specimen’s fracture are recorded.
(3) We observe whether the specimen with the built-in repair system has any adhesive flow out after loading, make a record, and then gently place it to undergo self-healing.
(4) We compare the cracking load values of the two types of specimens to determine the effect of the built-in repair system on the performance of concrete, and calculate the strength loss rate.
(5) We subject the self-healing system to secondary loading and record the respective failure locations as well as the failure loads.

After the first three-point bending test was completed, the specimens were left to stand for 7 days to fully cure and reach adhesive strength before we proceeded with the second three-point bending test. The same loading protocol as the first test was adopted for the repaired components to allow for comparative analysis. During the second three-point bending test, observations were made, not only of the cracks generated from the first test but also for the emergence of any new cracks. It was important to observe whether the cracks from the initial test would reopen and to monitor the location of cracks generated during the second test, as well as to check whether adhesive would flow out from the sites of new cracks.

## 3. Results and Discussion

### 3.1. Adhesive Formulation

The results of nine experiments indicated that the most ideal outcome was achieved in the second trial, where the optimal group was identified with a formulation consisting of 15 g of 692 diluents, 100 g of 650 curing agents, and 30 g of acetone diluent. This formulation demonstrated a load-bearing capacity of 256.175 N and a flexural strength of 0.84 MPa. The initial flexural strength of the two control cement mortar specimens was 2.33 MPa, resulting in a strength recovery rate of 36.1% after the repair process. The results are presented in Table 8.

### 3.2. Flow and Curing Effect of Epoxy Adhesives

The experimental results indicate that while the addition of diluents improves the fluidity of the epoxy resin, it also affects the curing effect, resulting in a flexural strength recovery rate that does not meet expectations. Firstly, the optimal formulation ratio is selected for viscosity testing using a six-speed rotational viscometer. The correct rotational speed should be chosen to ensure that the experiment is conducted under conditions that maintain a steady flow of the liquid at a constant temperature. The adhesive being tested must be thoroughly mixed. The mixture of 650 low-molecular-weight polyamide and acetone must be adequately stirred, and since acetone is volatile, the experiment should be conducted promptly after achieving a uniform mixture. The viscosity of adhesives at different dilution ratios is shown in Table 9.

The viscosity of the adhesive must be reduced to below 20 MPa·s to meet the experimental requirements. Based on the data obtained at a rotation speed of 3 r/min, this set of proportions meets the condition. Experiments have shown that when the amount of acetone does not exceed 40 g, the curing time for the epoxy resin adhesive system is between 3 and 4 days, with the full curing strength reaching its maximum value around a week. When more than 50 g of acetone is added, the epoxy resin adhesive system is essentially impossible to cure. Through curing experiments, it is concluded that the amount of the non-reactive diluent acetone should not be excessive; otherwise, it will affect the normal curing of the adhesive.

Based on the established effective strength prediction formula in conjunction with Table 6: $S=S_{o}(1-f_{p}^{\frac{2}{3}})$, theoretical strength loss rate is presented in Table 10:

The effect of glass spheres on the compressive strength of the concrete matrix is observable, with a weakening effect that is greater than any strengthening effect, resulting in a certain rate of strength loss. The flexural strength loss rate of the experiment is displayed in Table 11. However, the impact is not significant within a dosage of 6%. The theoretical strength loss rate is greater than the experimental value, which may indicate that the predictive model does not account for the positive influence of the glass material on the concrete’s strength.

There is no positive influence of glass spheres on the flexural strength of the concrete matrix, and the weakening effect is quite pronounced. The reason for this may be the poor flexural performance of the glass spheres; when present in large quantities, the flexural strength of the entire concrete specimen can be significantly limited by the spheres. Observations from the fracture surface indicate that the glass spheres essentially remain intact, suggesting that cracks failed to penetrate through the spheres successfully. The use of glass spheres as the resin container is not advisable.

### 3.3. Influence of Glass Tube Volume Fraction on Concrete Flexural Strength

Hollow glass tubes have a tensile strength higher than that of concrete. In reinforced concrete without steel rebars, hollow glass tubes can, to a certain extent, serve the function of reinforcement, bearing a portion of the tensile stress. From this perspective, hollow glass tubes can improve the strength of concrete to a certain degree. Theoretical calculation yields a critical volume fraction of 7% for glass tubes. However, experiments conducted under the guidance of this theoretical data revealed that the actual flexural strength loss rate reached 31.3%, indicating that at a volume fraction of 7%, the reduction in concrete’s inherent strength due to the glass tubes was already significant. Based on this finding, this section carries out experiments on 12 cement mortar specimens, with 9 of them incorporating hollow glass tubes and the remaining 3 serving as conventional flexural mortar specimens. The experiments ultimately determine the flexural strength loss rate to ascertain the appropriate volume fraction. The data are presented in Table 12.

The experimental results indicate that the volume fraction of hollow glass tubes used for repair, when kept within 2%, does not significantly affect the flexural load-bearing capacity of mortar specimens. With a minimal decrease in the specimens’ flexural load-bearing capacity, incorporating more tubes increases the amount of adhesive, thereby enhancing the repair effect. It is suggested that the volume fraction of the glass tubes should be limited to 2% of the total volume of the specimens.

This study selected two short quartz glass tubes, each with an inner diameter of 10 mm, a wall thickness of 1 mm, and a length of 40 mm, for end-to-end joining. The connection was sealed with glass adhesive, and the experimental results indicated that the sealing at the joint was relatively good. It is anticipated that cracks will penetrate through the joint, releasing the adhesive to complete self-healing. Meanwhile, the dual channels ensure the storage and successful mixing of the two-component adhesive after release. A three-point bending test was conducted on cement mortar specimens, and when the loading value approached the limit range, the area about to crack was carefully observed in order to stop loading in time before the crack width exceeded 2 mm. Observations were made to record whether the built-in repair system specimens had adhesive flow after loading. After the three-point bending test on the specimens with the built-in self-repair system, the adhesive successfully flowed out, and the crack penetrated through the glass tube. This paper determined that the resin storage container should be a hollow glass tube (dual-channel variant).

### 3.4. Self-Healing Epoxy Resins and Curing Agent Mixing Issues in Self-Repair Mode

The epoxy resin and curing agent struggle to mix sufficiently under self-healing conditions, which affects the healing effect. The experimental results are shown in Table 13.

Comparing the results of the two experiments, the average flexural strength recovery rate after the second test was 20.3% for Group A and 24.9% for Group B. The higher flexural strength recovery rate in Group B suggests that the cracks generated during the flexural test primarily initiated from the bottom of the specimens. Therefore, embedding the healing agent closer to the bottom position yields better repair effects.

After the second three-point bending test, upon completely compressing the specimens, it was observed that the adhesive had mostly flowed out, but a small amount of curing agent remained at the bottom of the tubes. Incomplete mixing and curing reaction without manual stirring are important reasons for the low strength recovery rate. The diluent affected the curing rate and bonding effect. After the second test of the self-healing experiment, where the specimens were crushed, it was found that a small amount of adhesive was not fully cured, and the diluent also impacted the repair effect of the concrete. The flexural strength loss rate of self-healing concrete specimens is presented in Table 14.

Working according to Equations (11), the flexural strength loss rates for Groups A and B were calculated. A comparison among the three experimental groups reveals that the self-healing system exerts a certain diminishing effect on the flexural strength of concrete. Specimens in Groups A and B, which are embedded within a self-healing system, feature distinct embedding positions. The data from the initial loading test suggest that Group B’s diminishing effect on the concrete specimens’ flexural strength is more pronounced than that of Group A. This is attributed to the increased presence of hollow glass tubes at the base of the specimens, which elevates the concrete’s porosity and results in a more significant reduction compared to Group A. Experimental observations indicate that for both Groups A and B, the fracture and failure during the second loading predominantly occurred at the crack sites from the first loading, specifically within the adhesive layer post-repair. This type of failure is characterized as interfacial, with the cracks continuing to propagate along the adhesive surface. It can be deduced that the failure of self-healing concrete is attributable to the fracture of the adhesive layer, identifying the interfacial layer as the most vulnerable component.

### 3.5. Concrete Damage Analysis

**Figure 9:**
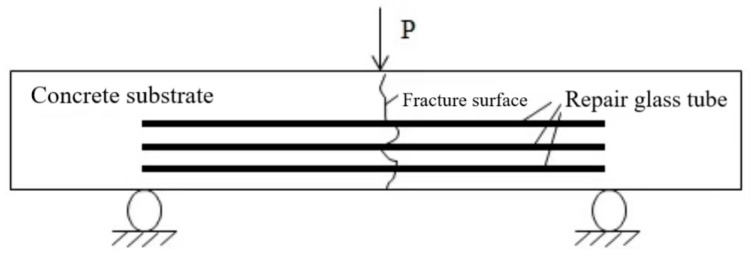
Crack propagation diagram during first loading.

**Figure 10:**
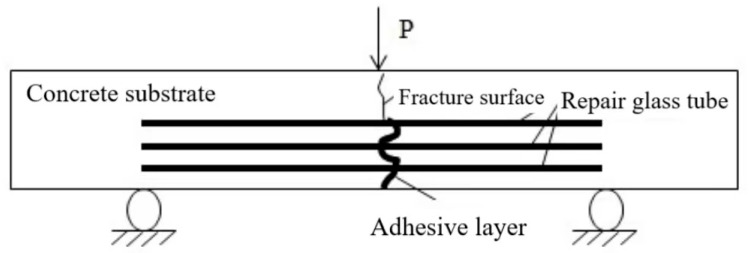
Crack propagation diagram during second loading.

The experimental phenomena indicate that during the second three-point bending test, the majority of the cracks occurred at the sites of the initial cracks from the first loading test. The adhesive layer became the weak link, with its fracture strength significantly lower than seen during the first test. The characteristics of failure in the second test are as follows: The fracture predominantly occurs at the adhesive surface. With the increase in load, the deflection at the specimen’s central point and the crack mouth displacement also continuously increases. The strain of the concrete along the height of the crack also increases, and the subcritical crack extension at the crack tip steadily increases. As the load approaches the point of failure, the subcritical crack at the tip undergoes unstable propagation, forming a macroscopic crack that rapidly penetrates along the adhesive surface, leading to specimen fracture. Observations during the test reveal that the fracture trajectory line of the second loading is consistent with that of the first loading, with cracks predominantly developing along one side of the adhesive layer, resulting in adhesive debonding. A typical fracture is depicted in Figure 9 and Figure 10.

From the experimental results, it can be observed that after the second loading test, the majority of the cracks occurred at the adhesive bonding surface, which is indicative of interfacial fracture failure. The cracks propagated along the bonding surface rather than within the concrete matrix. Only in a few specimens did the cracks develop within the concrete matrix upon failure.

### 3.6. Existing Problems

The adhesive used in the concrete repair system exhibited a lower healing rate than expected, with certain deficiencies observed in its release and curing reaction after mixing. During the experiments, no flow of adhesive was observed in some groups with crack widths around 2 mm, and only when the load was increased to produce larger crack widths did the flow phenomenon occur. This was ineffective for the repair of minor cracks. After the concrete was repaired, a secondary three-point bending test was conducted, revealing that in some groups, several minor cracks had formed again on top of the original main cracks, and these cracks were difficult to repair again with the existing adhesive.

### 3.7. Future Research Directions

Although many researchers at home and abroad have conducted relevant studies on the self-healing technology of concrete cracks and have made certain progress, there are still many issues that have not been resolved and require further research: the amount of adhesive depends on the quantity of the resin carrier. From the perspective of achieving self-healing effects, the more adhesive, the better. However, considering the maintenance of the concrete’s own properties, the amount of storage carrier should be controlled within an appropriate range. Therefore, determining the optimal amount of resin carrier is a process of weighing the pros and cons, and the choice of resin storage containers still deserves in-depth study. In the absence of external forces, the flow of adhesive is relatively slow and fails to mix fully for a complete curing reaction, affecting the healing effect. It is necessary to continue searching for a more suitable adhesive as a healing agent and to study the control and release of the healing agent to achieve better healing effects. It is also possible to explore a heating system to promote the reaction of the adhesive and improve the healing effect. The glass double tubes used in this paper do not provide good feedback for crack penetration; they fail to release the adhesive in time when minor cracks occur. Although there is theoretical guidance, a large number of comparative experiments on parameters such as wall thickness, tube diameter, and length are still needed to find the optimal size. In addition to this, only the flexural strength before and after the repair of self-healing concrete is tested and the recovery rate is calculated to evaluate the healing effect, without further research on the durability of self-healing concrete. It is hoped that when conditions are ripe in the future, this research can continue.

## 4. Conclusions

This article presents a three-point bending test conducted on the entire self-healing system, utilizing C30 concrete as the matrix, glass double tubes with a length of 80 mm, a wall thickness of 1 mm, and an inner diameter of 10 mm as the resin storage containers, and modified epoxy resin as the healing agent. The experimental data indicate that the healing system has a certain strength recovery effect on the concrete matrix, but the rate of strength recovery is not high, and there are still areas for improvement. The main conclusions of this paper are as follows:

1. Double-channel hollow glass tubes can serve as resin storage containers for the self-healing of concrete.
2. Considering the operational principle of self-healing concrete, an epoxy resin adhesive with dual-component features was chosen. The E-51 epoxy resin, when mixed with 692 reactive diluents at an appropriate proportion at room temperature, can decrease the viscosity and enhance the fluidity. Orthogonal experiments have established the formulation ratio of epoxy resin, 692 reactive diluent, 650 low-molecular-weight polyamide curing agent, and acetone as follows: 100:15:100:30.
3. Applying the critical volume fraction theory and examining the working mechanism of glass tubes in concrete based on the equilibrium state of the differential elements of hollow glass tubes, the critical volume fraction for embedding repair glass tubes was theoretically determined to be 7%, with a critical length of 40 mm and a wall thickness of 1 mm.
4. Using C30 concrete as the base material with embedded glass double tubes at a volume fraction of 2%, a two-component adhesive was employed to conduct a three-point bending test on the entire repair system. The results indicated that the average flexural strength recovery rates were 20.3% for Group A and 24.9% for Group B.

The results did not meet the expected outcomes. We observed that the diluents added to increase the fluidity of the epoxy resins and curing agents, to some extent, affected the curing effect; hence, further research on the formulation is warranted. The epoxy resins need to be thoroughly mixed with the curing agents. Under the self-healing mode, the two were difficult to react completely, which impacted the healing effect.

## Figures and Tables

**Table 1:** Orthogonal test design table.

Serial No.	W (692 Diluent)	W (650 Curing Agent)	W (Acetone)
1	15	90	25
2	15	100	30
3	15	110	35
4	20	90	30
5	20	100	35
6	20	110	25
7	25	90	35
8	25	100	25
9	25	110	30

**Table 2:** Acetone composition.

Serial No.	Epoxy Resin (g)	Reactive Diluent (g)	Curing Agent (g)	Acetone (g)
1	100	15	100	0
2	100	15	100	20
3	100	15	100	30
4	100	15	100	40
5	100	15	100	50
6	100	15	100	60

**Table 3:** Proportions of 12 sets of cement mortar test blocks.

Cement (g)	Water (kg)	Sand (kg)
1.8	0.9	5.4

**Table 4:** Concrete mix proportions.

Water-to-Binder Ratio	Cement (kg/m^3^)	Water (kg/m^3^)	Sand (kg/m^3^)	Stone (kg/m^3^)
0.47	394	185	571	1250

**Table 5:** Theoretical compressive strength loss rate.

Group	Loss Rate (%)
1	0
2	7.3
3	11.7
4	15.3

**Table 6:** Experimental compressive strength loss rate.

Group	Volume Ratio	Max Load (kN)	Compressive Strength (MPa)	Loss Rate (%)
1	0%	3.80	3.80	0
2	2%	3.57	3.57	6.1
3	4%	3.51	3.51	7.6
4	6%	3.42	3.42	10

**Table 7:** Experimental flexural strength loss rate.

Group	Volume Ratio	Max Load (kN)	Flexural Strength (MPa)	Loss Rate (%)
1	0%	1.06	3.49	0
2	2%	0.71	2.33	33.2
3	4%	0.72	2.37	32.1
4	6%	0.65	2.14	38.7

**Table 8:** Experimental results.

**Serial No.**	**Load-Bearing Capacity (N)**	**Bending Strength (MPa)**
1	135.214	0.44
2	256.175	0.84
3	151.886	0.50
4	146.612	0.48
5	169.259	0.56
6	155.507	0.51
7	161.615	0.53
8	203.221	0.67
9	155.024	0.51

**Table 9:** Viscosity of adhesives at different dilution ratios (MPa·s).

	Rotational Speed (r/min)	3
Dilution Ratio		
Epoxy Resin: 692 Diluent (1:0.15)		16.0
650 Curing Agent: Acetone (1:0.3)		19.5

**Table 10:** Compressive strength loss rate of experiment.

Experimental Group	Maximum Load (KN)	Compressive Strength (MPa)	Loss Rate (%)
1 0%	3.80	3.80	0
2 2%	3.57	3.57	6.1
3 4%	3.51	3.51	7.6
4 6%	3.42	3.42	10

**Table 11:** Flexural strength loss rate of the experiment.

Experimental Group	Maximum Load (KN)	Flexural Strength (MPa)	Loss Ratio (%)
1 0%	1.06	3.49	0
2 2%	0.71	2.33	33.2
3 4%	0.72	2.37	32.1
4 6%	0.65	2.14	38.7

**Table 12:** Flexural strength loss rate of cement mortar test blocks with glass fiber reinforcement.

Geometric Parameters of Glass Tubes	Specimen Number	Number of Glass Tubes	Volume Ratio	Flexural Strength (MPa)	Average Flexural Strength (MPa)	Flexural Strength Loss Rate
Inner diameter 10 mm Wall thickness 1 mm Length 40 mm	1	1	1.8%	2.71	2.56	13.9%
	2			2.33		
	3			2.65		
	4	2	3.5%	2.41	2.40	19.1%
2	5			2.52		
	6			2.29		
	7	4	7.1%	1.84	2.04	31.3%
	8			2.13		
2	9			2.17		
Without glass tubes	10	0	0	3.08	2.97	0
	11			3.27		
	12			2.57		

**Table 13:** Flexural strength of self-healing concrete before and after repair.

Parameter		Placement Location	Specimen Number	Pre-Repair Flexural Strength (MPa)	Post-Repair Flexural Strength (MPa)	Flexural Strength Recovery Rate	Average
Glass Tube	Adhesive						
Diameter 10 mm	Modified epoxy resin adhesive	Evenly distributed	A1	4.44	0.88	19.8%	20.3%
			A2	3.92	0.84	21.4%	
Length 80 mm			A3	4.02	0.79	19.7%	
		Near the base	B1	3.83	1.07	27.9%	24.9%
Quantity 8 pieces			B2	4.40	1.02	23.2%	
			B3	3.64	0.86	23.7%	
Non-repaired glass tubes			C1	5.17			
			C2	4.42			
			C3	4.59			

**Table 14:** Flexural strength loss rate of self-healing concrete specimens.

Parameter		Placement Location	Specimen Number	Pre-Repair Flexural Strength (MPa)	Average	Flexural Strength Loss Rate
Glass Tube	Adhesive					
Diameter 10 mm	Modified epoxy resin adhesive	Evenly distributed	A1	4.44	4.13	12.5%
			A2	3.92		
Length 80 mm			A3	4.02		
		Near the base	B1	3.83	3.96	16.1%
Quantity 8 pieces			B2	4.40		
			B3	3.64		
Non-repaired glass tubes			C1	5.17	4.72	--
			C2	4.42		
			C3	4.59		

## Data Availability

The data used in the article are reflected in the article.
